# Supplementary material for: Heritability of complex traits in sub-populations experiencing bottlenecks and growth
Source: J Hum Genet. 2024 Apr 8;69(7):329–35. doi: 10.1038/s10038-024-01249-2 (PMC11199143; doi:10.1038/s10038-024-01249-2)
Supplement: Supplementary file 1 — Supplemantal Material [file 10038_2024_1249_MOESM1_ESM.docx]

# Supplementary Information for Heritability of complex traits in sub-populations experiencing bottlenecks and growth

Cameron S. Taylor and Daniel J. Lawson

The effective amount of genome simulated can be controlled by scaling the simulation parameters, though the scaling is not completely trivial. By changing the recombination rate, sequence length and mutation rate together, we can control the genome wide genomic architecture. For the purpose of this model it is equivalent to changing the polygenicity (i.e. the proportion of SNPs that have an effect on the trait) since in this model all SNPs that are simulated are treated as causal.

Following Kelleher et al., 2016 and Ogundijo and Wang, 2017, the expectation is that if the effective recombination rate, *sequence length* ×*recombination rate* = *constant* and the relative mutation rate, *mutation rate/recombination rate* = *constant*, then the genomic architecture should be equivalent. This follows from the definitions of the scaled recombination rate *ρ* = 4*N_e_L* and the scaled mutation rate *θ* = 4*N_e_µ*.


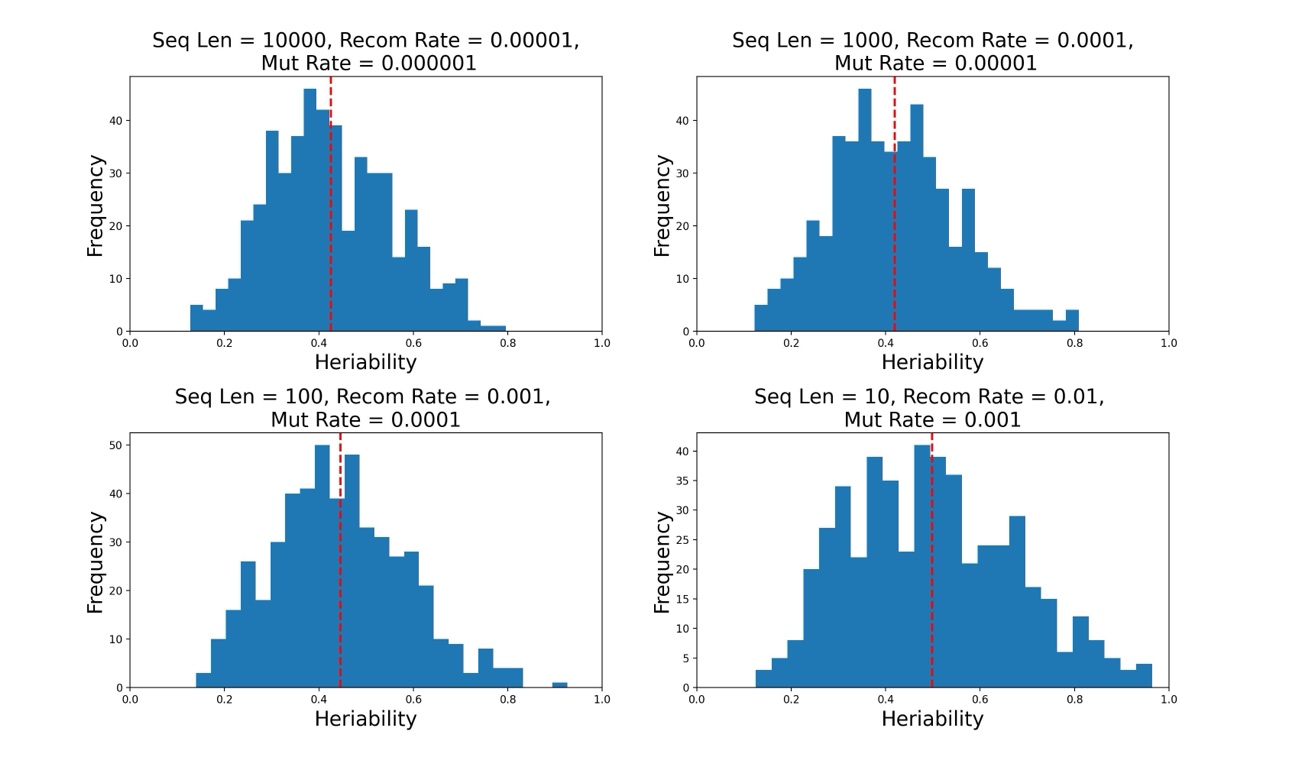


Figure S1: Heritability distribution across scaled sequence length, recombination rate and mutation rate that model the same genetic architecture. (a) sequence length = 1*e*4, recombination rate 1*e*−5, mutation rate = 1*e*−6, (b) sequence length = 1*e*3, recombination rate = 1*e*−4, mutation rate = 1*e* − 5, (c) sequence length = 1*e*2, mutation rate = 1*e* − 3, mutation rate = 1*e* − 4, (d) sequence length = 10, recombination rate = 0.01, mutation rate = 0.001.

Figure S1 shows 500 repeated experiments in four different combinations of heritability and sequence length with *Lρ* = 0*.*1 and *µ/ρ* = 0*.*1. The distribution of heritability remains close to consistent in all four cases, though there may be increased volatility as the sequence length is decreased. However, Figure S2 shows the outcome of the experiments with different *recombination rate*, which despite sharing the same expected heritability, produces a different distribution. The variance in heritability increases, as we have decreased the effective number of independent genome regions and hence increased autocorrelation along the genome. In this sense, both polygenicity and recombination rate are parameters of genomic architecture.


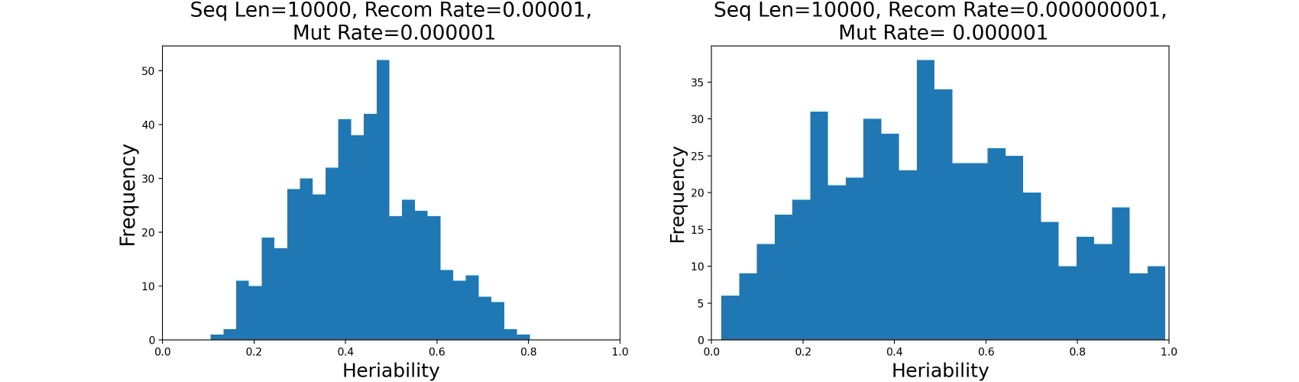


Figure S2: Heritability distribution in differing genetic architectures. (a) sequence length = 1*e*4, recombination rate = 1*e* − 5, mutation rate = 1*e* − 6, (b) sequence length = 1*e*4, recombination rate = 1*e* − 8, mutation rate = 1*e* − 6 (i.e. *ρ* is scaled by 0*.*001).
